# The relationship of gray zone and infarct core in the Iceland MI study

**DOI:** 10.1186/1532-429X-18-S1-P99

**Published:** 2016-01-27

**Authors:** Vishal C Mehra, Li-Yueh Hsu, Christopher Miller, Andrew E Arai

**Affiliations:** 1 grid.415341.60000000404334040Cardiology, Geisinger Clinic, Danville, PA USA; 2 grid.94365.3d0000000122975165NHLBI, National Institutes of Health, Bethesda, MD USA

## Background

Gray zone or infarct heterogeneity, defined as the admixture of necrotic and viable myocardium at the periphery of myocardial infarction (MI), has been noted to be a modulator of cardiac outcomes. Most studies have not reported a direct correlation between infarct core and the zone of heterogeneity. Furthermore, no large population based studies have examined the burden of undiagnosed infarct and infarct heterogeneity.

## Methods

We report the data from 215 individuals (of the total 950 enrolled in the Iceland MI study) who were found to have myocardial infarction by late gadolinium enhancement on CMR. The infarct core and gray zone assessment was performed by FWHM and 2SD threshold, respectively.

## Results

The median infarct core, as a percentage of left ventricular mass was 6.3% (5.4-6.9, 95% CI) and the gray zone was 4.5% (3.8-5.2, 95% CI). The correlation between gray zone and infarct core assessed by Spearman's rank coefficient (rho) was 0.88 (0.80-0.90) P < 0.0001. When gray zone was normalized to total scar size, it had a negative correlation with infarct core, rho of -0.53 (-0.62--0.42), P < 0.0001. Furthermore, non-linear regression with a simple power function fit the relationship between gray zone and infarct size (R^2^ = 0.74).

## Conclusions

These data suggest that gray zone correlates strongly to infarct core size but the relationship is best explained by a nonlinear regression model. Gray zone constitutes a larger portion of total scar in smaller infarcts. Further analysis of clinical outcomes will shed light on the differential prognostic information provided by these CMR parameters.

**Figure 1:**
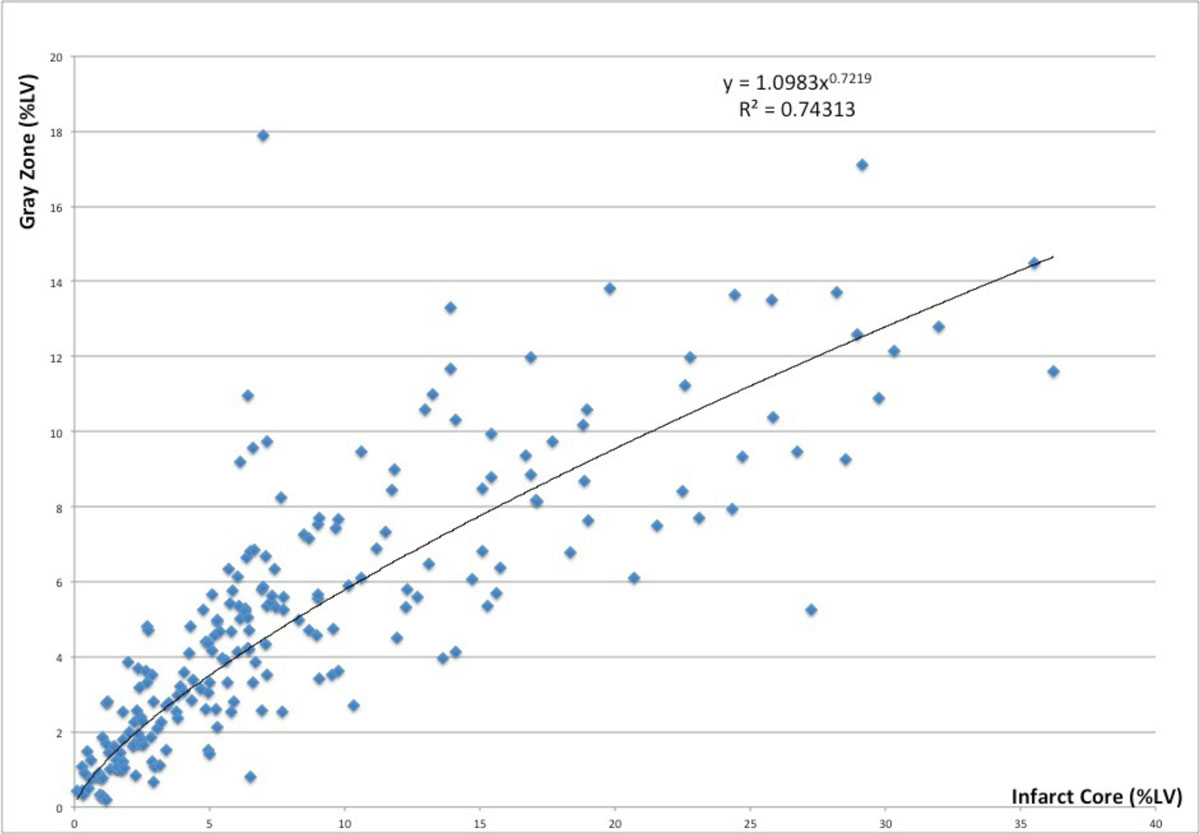
**Nonlinear regression with a power function fits the relationship between gray zone and infarct core (R**^**2**^ **= 0.74)**.

